# Delayed ICU admission with community-acquired severe sepsis greatly increases mortality and resource use

**DOI:** 10.1186/cc9631

**Published:** 2011-03-11

**Authors:** A Shorr, Y Choe, W Linde-Zwirble

**Affiliations:** 1 Washington Hospital Center, Washington, DC, USA; 2 Eisai Inc., Woodcliff Lake, NJ, USA; 3 ZD Associates LLC, Perkasie, PA, USA

## Introduction

While many severe sepsis (SS) patients go to the ICU on hospital admission, others with community-acquired infection (CAI) either progress to SS later in the hospitalization or are not considered severely ill on admission. The proportion of SS cases falling into these two groups is not known, and their outcomes are not well described.

## Methods

We identified all adult hospitalizations in the 2008 Premier database that had an ICD-9-CM code for SS (995.92, 785.52), a CAI, and who entered the hospital through the ED (for example, not transferred from another hospital). Patients were characterized by the sequence of ICU and floor care, the number of antibiotic classes (AbxC) on day 1, and the duration of floor stay before ICU admission. We assessed resource use via length of stay (LOS) and total cost. We also examined hospital mortality.

## Results

The cohort included 33,059 discharges (49.1% male, mean age 69.0 years), of whom 17,690 (53.5%) were admitted to the ICU at hospital presentation. Mortality in direct to ICU subjects equaled 31.2%, and these patients had an average LOS of 12.0 days with a mean cost of $30,174, with only 22.8% given a single AbxC. Those admitted to the floor initially (46.5%) had a similar LOS (11.7 days) and mortality (31.1%) but had lower mean costs ($22,728) and nearly half (49.3%) had a single AbxC. Of these initial floor patients, those that were never admitted to the ICU (28.0% of all cases) had the shortest stay (7.6 days), lowest cost ($11,753), and lowest mortality (24.2%) with 44.3% receiving a single AbxC on day 1. Those starting on the floor and later transferred to the ICU (18.4% of all cases) had the longest stay (17.7 days), highest cost ($39,332) and highest mortality (41.5%), and were most likely to have a single AbxC on day 1 (56.8%). Even those admitted to the ICU after 1 day on the floor (3,179, 52.1% of delayed ICU cases) had higher mortality (36.0%) than those starting in the ICU (*P *< 0.0001). Mortality increased with longer delays before ICU admission (40.7%, for a 2-day delay (14.1% of delayed cases) and 50.3% for those with a 3-day or more delay (33.8% of delay cases)).

## Conclusions

SS patients with CAI admitted to the floor and later transferred to the ICU are a major fraction of all SS cases and have the worst outcomes. While many may have developed organ dysfunctions later in the hospitalization, nearly two-thirds were admitted to the ICU after just 1 or 2 days on the ward, indicating that they may have been mis-triaged. Interventions to better identify and aggressively treat these cases may improve outcomes.

